# Analyzing Key Factors Influencing Water Transport in Open Air-Cooled PEM Fuel Cells

**DOI:** 10.3390/ma17133267

**Published:** 2024-07-02

**Authors:** Bin He, Lin Wei, Fengping Hu, Ahmed Mohmed Dafalla, Jian Guo, Cuihua Wang, Fangming Jiang

**Affiliations:** 1 Guangzhou Institute of Energy Conversion, Chinese Academy of Sciences, Guangzhou 510640, China; hbinyeah@163.com (B.H.); hufp@ms.giec.ac.cn (F.H.); mohned@ms.giec.ac.cn (A.M.D.); guojian@ms.giec.ac.cn (J.G.); 2 School of Mechanical and Power Engineering, Shenyang University of Chemical Technology, Shenyang 110142, China; wch-7855@163.com; 3 CAS Key Laboratory of Renewable Energy, Guangzhou 510640, China

**Keywords:** proton exchange membrane fuel cell, air-cooled, water and heat management, uniformity, numerical analysis

## Abstract

The current limitations of air-cooled proton exchange membrane fuel cells (AC-PEMFCs) in water and heat management remain a major obstacle to their commercialization. A 90 cm^2^ full-size AC-PEMFC multi-physical field-coupled numerical model was constructed; isothermal and non-isothermal calculations were performed to explore the effects of univariate and multivariate variables on cell performance, respectively. The isothermal results indicate that lower temperature is beneficial to increase the humidity of MEA, and distribution uniformity at lower stoichiometric ratios and lower temperatures is better. The correlation between current density distribution and temperature, water content, and concentration distribution shows that the performance of AC-PEMFCs is influenced by multiple factors. Notably, under high current operation, the large heat generation may lead to high local temperature and performance decline, especially in the under-channel region with drier MEA. The higher stoichiometric ratio can enhance heat dissipation, improve the uniformity of current density, and increase power density. Optimal fuel cell performance is achieved with a stoichiometric ratio of 300, balancing the mixed influence of multiple factors.

## 1. Introduction

In the context of addressing the dual pressure of global energy demand and environmental protection, the development of clean energy technology is particularly important. Proton exchange membrane fuel cells (PEMFCs) have garnered significant attention for their ability to efficiently convert chemical energy directly into electrical energy through electrochemical reactions [1,2], and they are widely used due to their high energy density and low environmental impact. Currently, fuel cells are utilized in various applications, including transportation, stationary power generation systems, and portable power sources [3,4,5,6]. Compared with liquid-cooled fuel cells, air-cooled proton exchange membrane fuel cells (AC-PEMFCs), which use fans to supply air for both reaction and cooling, are especially favored in small equipment and low-power applications due to their simpler systems and lower costs [7,8,9]. However, AC-PEMFC has adjustable variables, and the air stoichiometric ratio/air flow rate affects its performance in several ways [10]. A high stoichiometric ratio enhances water evaporation and results in low ionic conductivity, which in turn affects the performance and lifetime. Conversely, a low stoichiometric ratio will cause an insufficient supply of reactants, which will directly increase polarization loss and raise the internal resistance of the electrodes [11]; additionally, at low flow rates, the generated water cannot be discharged in time, leading to flooding [12].

The air flow rate is directly related to the heat dissipation of AC-PEMFC. The sources of heat in PEMFC include electrochemical reaction heat and ohmic heat, and the heat generation increases with the current density. Luo et al. [13] conducted experiments on the temperature characterization of AC-PEMFCs and found that the temperature difference on the outer surface of the fuel cell stack increases from 5.2 °C to 12.9 °C as the current increases. Chang et al. [14] found that the maximum and average temperatures of the air-cooled stack exhibit a “parabolic” distribution, with the maximum temperature difference between the individual cells at the cathode inlet side also displaying a “parabolic” distribution. However, the temperature difference at the cathode exit side was “anti-parabolic”. The temperature difference between different single tanks and within the single tanks could reach 19.7 °C and 8.4 °C, respectively. If the heat generated by PEMFC cannot be dissipated in time, it may generate localized overheating points [15], leading to MEA damage [16].

The water balance in a fuel cell is very sensitive to temperature. High temperature will accelerate evaporation and lead to the dehydration of MEA (membrane electrode assembly), while low temperature may lead to flooding [17]. It is important to note that the cathode stoichiometric ratio typically needs to be greater than 50 in order to ensure a suitable operating temperature [18]. Peng et al. [19] found that when the cathode stoichiometric ratio increases from 30 to 230, the temperature of the cathode catalytic layer decreases by nearly 24 K. Zhang et al. [20] used a non-isothermal multiphase numerical model to simulate the electrochemical reactions and the heat and mass transfer processes in the stack, finding that an increase in the stoichiometric ratio facilitates the discharge of the waste heat from the stack and enhances the water content of the proton exchange membrane (PEM). Wei et al. [21] conducted experimental tests and numerical analyses on a self-assembled 800 W AC-PEMFC stack and found that increasing the air flow rate can effectively improve temperature uniformity in the stack under high current conditions. However, if the air flow rate is too high, it will lead to a higher water evaporation rate, potentially causing MEA dehydration again [22,23]. Hu et al. [24] found that water flooding becomes more severe with an increasing air stoichiometric ratio, leading to an uneven current density distribution and performance degradation. Similarly, Wang et al. [25] investigated the effect of load current on the temperature uniformity of the outer surface of the stack by modeling the temperature with the help of thermal imaging and found that, at low load currents, the internal temperature of the stack is low and the high liquid water content inside the stack affects the proton transport. These findings indicate that the interaction between water, temperature, and flow rate is an important factor in the study of AC-PEMFC performance. Further research and exploration are significant for optimizing the performance and lifetime of AC-PEMFC.

In order to achieve efficient water and heat management of AC-PEMFC, researchers have proposed control strategies and structural designs. Since the cathode reaction gas of AC-PEMFC is supplied by fans, different air supply methods impact the output performance of PEMFC stacks differently. Chen et al. [26] studied two air supply modes, blowing and exhausting, and found that when the ambient temperature is 30 °C, the output voltage can be increased by 5.5% in blowing mode compared with exhausting mode. It was found that the temperature in exhausting mode is slightly lower than that in blowing mode when the loading current is less than 30 A; however, when the current exceeds 30 A (temperature reaches 36 °C), the temperature in exhaust mode is higher than the temperature in blowing mode. Guo et al. [27] showed that, at low currents, a PWM (pulse width modulation) of 40% is ideal as it leads to higher temperatures and faster reaction rates, while at medium and high currents, a PWM of 60% is recommended to avoid overheating and flooding. Yuan et al. [28] constructed a three-dimensional numerical model to predict the temperature distribution of a fuel cell power stack in the cooling channels under different air flow rates. Thermal management control was implemented on a 1.2 kW fuel cell stack by adjusting the operating temperature to minimize the temperature gradient. The experimental results showed that the temperature gradient of the stack was reduced by 0.5 °C, significantly improving the performance. Wang et al. [29] proposed a real-time power optimization strategy based on active temperature control, providing a promising solution for the power optimization of AC-PEMFC in actual environments, especially in transportation where lightweight and small sizes are required. Mahjoubi et al. [30] designed a safe and optimal operating interval that is controlled by the load current, stack temperature, and air stoichiometric ratio. A new control strategy was proposed to regulate the stack temperature and air stoichiometric ratio simultaneously, and the operating efficiency was significantly improved.

Additionally, structural design optimization can improve heat dissipation, prevent cell overheating, and reduce energy loss. Zhang et al. [31] proposed a staggered tapered slot flow field, which increases the contact area of the air flow with the bipolar plates by arranging tapered and slotted surfaces in the channel along the air flow direction to enhance cooling. This design reduced the internal temperature of the mono cell by about 14.2–28.3 K and improved the MEA water content by about 35.1–85.796%. Geng et al. [32] designed a novel AC-PEMFC based on different cell aspect ratios (*R*_L/W_) and anode serpentine quantities. The results show that the uniformity of current density, oxygen mass fraction, temperature, and water can be improved by increasing *R*_L/W_. When *R*_L/W_ is small, the influence of anode number on performance and parameter distribution is greater than that of *R*_L/W_ of the bipolar plate. When *R*_L/W_ is greater than 2.94, the performance mainly depends on *R*_L/W_. Wei et al. [33] achieved improved performance by increasing the channel depth, increasing the rib–channel width ratio, and employing zigzag flow channels, highlighting the importance of air flow rate and temperature control to ensure optimized cell performance.

In summary, how the two factors, air flow rate and temperature, affect the performance of fuel cells and their interaction with water content is one of the hot issues in current research. Existing studies have mainly focused on the combined effects of flow rate and temperature on performance; however, the cause of the dehydration of MEA remains unclear. This study aims to clarify the interactions between air flow rate and temperature in AC-PEMFC and to explore optimal control strategies by using full-scale isothermal and non-isothermal numerical models. Through simulation analysis, the more critical influencing factors in the actual operating conditions will be revealed to provide a theoretical basis and technical support for the design and operation optimization of fuel cells.

## 2. Numerical Model

### 2.1. Computational Domain

**Figure 1:**
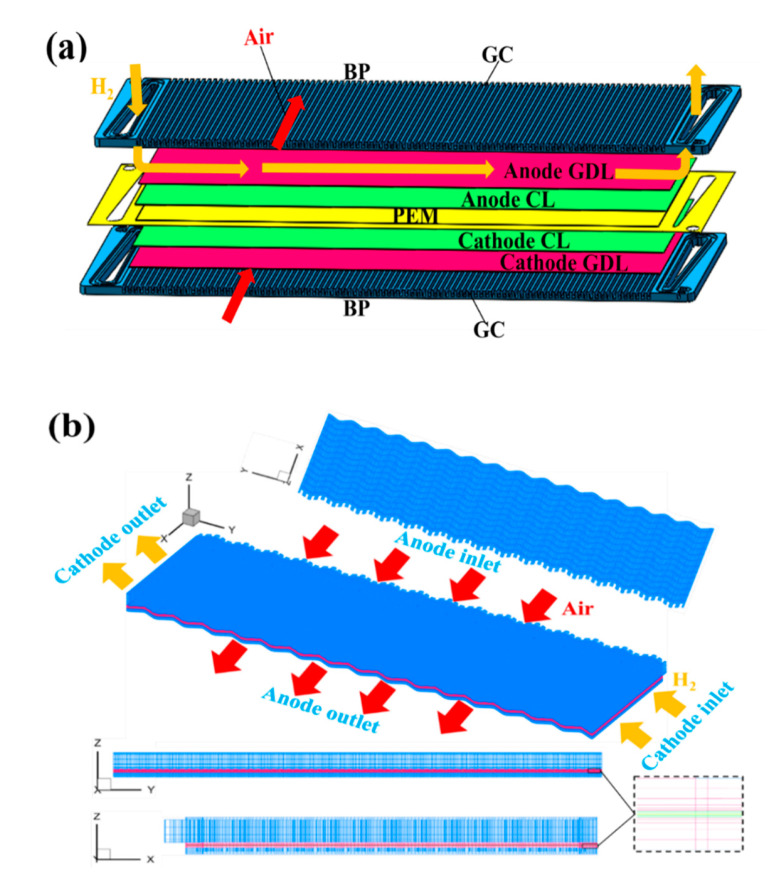
(**a**) 3D schematic of AC-PEMFC; (**b**) computational domain.

Figure 1a shows a schematic diagram of the three-dimensional structure of PEMFC, which consists of a bipolar plate (BP), a gas flow channel (GC), a gas diffusion layer (GDL), a catalytic layer (CL), and a proton exchange membrane (PEM). Parallel channels are inscribed on the cathode side of the bipolar plate, covering the cathode side of MEA to form the cathode gas flow field. Similarly, zigzag-shaped channels are inscribed on the anode side of the bipolar plate, covering the anode side of MEA to form the anode gas flow field. Hydrogen and air flow through the inlet of the anode and cathode channels, respectively. The reactants are then transported to the catalyst layer to participate in the electrochemical reaction. The unreacted gases and water generated by the reaction are discharged from the outlet. On the anode side, hydrogen is oxidized to produce protons, which subsequently move to the cathode through the proton exchange membrane. At the same time, the electrons generated at the anode are transferred to the cathode via the bipolar plates and external circuitry, resulting in the formation of an electric current. It is worth noting that the cathode flow channel of AC-PEMFC is open, and both sides of the cathode gas flow channel are directly connected with ambient air, which is supplied to the gas flow channel via fans.

In this paper, the AC-PEMFC whole-plate region is used as the computational domain, and a structured grid is adopted with a total of 2,835,200 cells. The computational domain grid is shown in Figure 1b. The structural parameters and operating conditions are shown in Table 1.

### 2.2. Governing Equations

The reactions carried out inside an AC-PEMFC influence each other. The following assumptions are made to ensure the premise of modeling accuracy and precision as much as possible [34]:

(1) It is assumed that the model is operated under dry conditions, ignoring the gas–liquid phase transition. There is no need to consider the presence of liquid water within the model, at which point the fluid motion is presented as a mist flow. According to this premise, a mild super-saturation of gas-phase moisture can be tolerated, because the initial dry gas flow will effectively carry away most of the moisture generated in PEMFC [35].
(2) Various processes within the cell will not vary over time or be affected by gravity.
(3) Gases are incompressible fluids, and this assumption is based on the ideal gas equation of state.
(4) Due to the small pressure gradient and Reynolds number, the state of fluid flow in the cell channels and porous media is considered laminar; i.e., the flow is smooth and without significant turbulence.
(5) A homogeneous medium model is used for describing material transport, and the properties of the medium are the same in all directions.

To describe the multi-physics transport and electrochemical processes within PEMFC during ambient operation, the four conservation equations used are as follows:

Mass continuity equation:

(1) $$\nabla \cdot \left(\rho u\right)=0$$

where *ρ* is the density of the gas mixture in kg/m^3^ and *u* is the velocity vector in m/s. The source terms arising from the generation and consumption of products and reactants within PEMFC during electrochemical reactions are neglected [36].

Conservation of momentum equation:

(2) $$\frac{1}{\varepsilon }\left[\frac{1}{\varepsilon }\nabla \cdot \left(\rho u^{2}\right)\right]=-\nabla P+\nabla \cdot \left(\mu \nabla u\right)+S_{u}$$

(3) $$S_{u}=-\frac{\mu }{K}u$$

where *ε* is the porosity, *P* is the pressure in Pa, and *μ* is the kinematic viscosity in Pa·s. In Equation (2), the effect of the porous layer on fluid transport is considered, including the momentum source term *S_u_*, and convective mass transfer in the PEM is neglected.

Energy conservation:

(4) $$\;\nabla \cdot \left(\rho c_{p}uT\right)=\nabla \cdot \left(k_{\mathrm{eff}}\nabla T\right)+S_{T}$$

where *T* is the local temperature, *c_p_* is the specific heat capacity, and *k_eff_* is the effective thermal conductivity in W/(m·K). *S_T_* denotes the heat source term, including reversible heat from entropy change, irreversible heat from the electrochemical reaction, and ohmic heat.

Charge conservation:

(5) $$0=\nabla \cdot \left(\kappa_{\mathrm{eff}}\nabla \phi_{e}\right)+S_{\phi_{e}}$$

(6) $$S_{\phi_{e}}=j_{a}$$

(7) $$0=\nabla \cdot \left(\sigma_{\mathrm{eff}}\nabla \phi_{s}\right)+S_{\phi_{s}}$$

(8) $$S_{\phi_{s}}=-j_{c}$$

where the effective proton conductivity of the MEA phase in the PEM and catalytic layer is denoted by *κ_eff_* and the effective electrode conductivity is denoted by *σ_eff_*. The rate of electron or proton production or consumption is denoted by $S_{\phi_{s}}$ and $S_{\phi_{e}}$, i.e., the source term. It can be expressed by the current density *j*, where *j_a_* and *j_c_* represent the anodic and cathode current densities, respectively. The kinetic equation of the electrochemical reaction can be described based on the following Butler–Volmer equation [37,38]:

(9) $$j_{a}=ai_{0,\mathrm{ref}}^{H_{2}}{\left(\frac{C^{H_{2}}}{C_{\mathrm{ref}}^{H_{2}}}\right)}^{1/2}\exp\left(\frac{\alpha_{a}+\alpha_{c}}{RT}F\eta \right)$$

(10) $$j_{c}=-ai_{0,\mathrm{ref}}^{O_{2}}\frac{C^{O_{2}}}{C_{\mathrm{ref}}^{O_{2}}}\exp\left(-\frac{\alpha_{c}}{RT}F\eta \right)$$

where the exchange current densities $ai_{0,\mathrm{ref}}^{H_{2}}$ and $ai_{0,\mathrm{ref}}^{O_{2}}$ depend on the hydrogen and oxygen concentrations in the baseline state in units of A/m^3^. The baseline hydrogen and oxygen densities are denoted as $C_{\mathrm{ref}}^{H_{2}}$ and $C_{\mathrm{ref}}^{O_{2}}$, respectively, in units of mol/m^3^, and the apparent transmission coefficients of the cathode and anode electrode reactions are denoted by *α_a_* and *α_c_*, respectively. *R* is the ideal gas constant, which is usually taken to be 8.314 J/(mol·K). The activation over-potential within the catalytic layer *η* is in the unit of V, and it can be calculated as follows:

(11) $$\eta =\phi_{s}-\phi_{e}-U_{0}$$

where the thermodynamic equilibrium potential *U*_0_ can be expressed as follows:

(12) $$U_{0}=1.23-0.0009\times (T-T_{0})$$

where *T*_0_ is the reference temperature. Changes in equilibrium potential due to changes in temperature and reactant concentration are neglected here.

Component conservation:

(13) $$\nabla \cdot \left(uC^{i}\right)=\nabla \cdot \left(D_{\mathrm{eff}}^{i}\nabla C^{i}\right)+S_{C}^{i}$$

In the reaction process of PEMFC, the key components include hydrogen, oxygen, water vapor, and water molecules stored in the electrolyte. The main regions involved are the anode and cathode flow channels, the diffusion layer, and the catalytic layer. To characterize the conservation of these components in each region, the standard form of the component conservation equation is usually used, as follows:

(14) $$S_{C}^{i}=-\frac{s_{k}j}{nF}-\nabla \cdot \left(\frac{n_{d}}{F}i_{e}\right)$$

where $S_{C}^{i}$ is the concentration source term for the component *k*, which represents the production and consumption of products or reactants within the catalytic layer and the electro-osmotic migration of water within MEA. $D_{\mathrm{eff}}^{i}$ is the effective diffusion coefficient considering the porosity, obtained by Bruggeman’s correction [39], as follows:

(15) $$D_{\mathrm{eff}}^{i}=D^{i}\varepsilon^{1.5}$$

Based on previous assumptions, the model assumes two forms of water presence: water vapor and membrane-bound water. For membrane-bound water, which is the water absorbed by electrolytes, it is represented by the water content, λ, defined as the content of membrane-bound water in the medium. It indicates the amount of water molecules associated with each mole of sulfate anion in the electrolyte, with units in mol/mol. The areas containing membrane-bound water are the CLs and PEM. According to the relationship between the water content λ and the water concentration,

(16) $$C_{m}^{H_{2}O}=\frac{\rho_{m}\lambda }{EW}$$

Since convection is only effective for water vapor in the pores, an expression for the relationship between the MEA water content λ and the water activity *a* can be written as follows [37]:

(17) $$\lambda =0.043+17.18a-39.85a^{2}+36.0a^{3}\left(0<a\;\leq \;1\right)$$

The water activity, *a*, is defined as follows:

(18) $$\;a=\frac{C^{H_{2}O}}{C_{\mathrm{sat}}^{H_{2}O}}$$

where $C_{\mathrm{sat}}^{H_{2}O}$ represents the saturated water vapor concentration at the current temperature; when *a* = 1, the corresponding water content is the maximum amount of water absorbed by the electrolyte *λ_sat_*.

In the PEM region, the diffusion coefficient of component water can be expressed as follows:

(19) $$D^{H_{2}O}=D_{m}^{H_{2}O}\frac{dC_{m}^{H_{2}O}}{dC^{H_{2}O}}=D_{m}^{H_{2}O}\frac{1}{EW}\frac{RT}{p_{\mathrm{sat}}}(\rho +\lambda \frac{d\rho }{d\lambda })\frac{d\lambda }{da}$$

where $D_{m}^{H_{2}O}$ and $D^{H_{2}O}$ are the diffusion coefficients of water within MEA and the diffusion coefficients of the hypothetical water concentration, respectively, and $dC_{m}^{H_{2}O}$ and $dC^{H_{2}O}$ are the amount of change of water within MEA and the amount of change of the hypothetical water concentration, respectively. Since the water-absorbing expansion of the electrolyte is not considered in this model, the electrolyte density is a constant value, and thus, $\frac{d\rho }{d\lambda }$ = 0.

In the CL region, the effective diffusion coefficient within the CL can be deduced and written due to the simultaneous presence of pores and electrolytes, combined with the above diffusion coefficients of the aqueous components within the pores and within the proton exchange membrane, as follows:

(20) $$D_{\mathrm{eff}}^{H_{2}O}=\varepsilon^{n}D^{H_{2}O}+{\left(\varepsilon_{m}\right)}^{n}D_{m}^{H_{2}O}\frac{dC_{m}^{H_{2}O}}{dC^{H_{2}O}}$$

where *ε_m_* is the volume fraction of electrolyte. The electrochemical and transport parameters are tabulated in Table 2.

To evaluate the uniformity of parameter distribution inside the fuel cell, the uniformity index is defined as follows [42]:

(21) $$NUI=\frac{\int_{A}\left|N_{\mathrm{loc}}-N_{\mathrm{avg}}\right|dA}{\int_{A}dA}$$

(22) $$N_{\mathrm{avg}}=\frac{\int_{A}NdA}{\int_{A}dA}$$

where *N* represents a parameter such as temperature or concentration, *N_loc_* is the value of the parameter in a certain location, *N_avg_* is the average value of the parameter, and *A* is the effective response area, m^2^. A lower index value indicates a higher uniformity.

### 2.3. Boundary Conditions

Velocity inlet and pressure outlet are used for the inlet and outlet of the gas flow channel, respectively. The velocity in the gas flow channel is determined based on the reference current density *I_ref_* and stoichiometric ratio *ξ* given during operation [21], as follows:

(23) $$u_{\mathrm{in},a}=\frac{I_{\mathrm{ref}}}{2F}\frac{\xi_{a}A_{\mathrm{PEM}}}{C_{\mathrm{in}}^{H_{2}}A_{a,\mathrm{GC}}},u_{\mathrm{in},c}=\frac{I_{\mathrm{ref}}}{4F}\frac{\xi_{c}A_{\mathrm{PEM}}}{C_{\mathrm{in}}^{O_{2}}A_{c,\mathrm{GC}}}$$

where $C_{\mathrm{in}}^{H_{2}}$ and $C_{\mathrm{in}}^{O_{2}}$ are the inlet hydrogen and oxygen concentrations, respectively, and *A*_a,GC_, *A*_c,GC_, and *A*_PEM_ are the anode gas inlet area, cathode inlet area, and proton exchange membrane area, respectively. Stoichiometric ratio is defined as the ratio between reactant feed and reactant consumption, and must always be greater than 1. Since the anode side is in “dead-end” mode, a low stoichiometric value (1.2) of hydrogen is calculated.

The cathode and anode inlets are set as velocity inlet boundaries. For both anode and cathode channels, the pressure outlet boundary conditions are used. The temperature boundaries of the anode and cathode gas inlets and the outside of the bipolar plate are set to the same constant value (ambient temperature). In the gas channel, the concentrations of H_2_, O_2_, and vapor at the gas channel inlet are set as constants determined by their respective pressures, relative humidity, and operating temperatures with the following expression:

(24) $$C_{\mathrm{in}}^{H_{2}}=\frac{\left(p_{a}-RH_{a}p_{\mathrm{sat}}\right)}{RT_{a}},\;C_{\mathrm{in}}^{O_{2}}=\frac{0.21\left(p_{c}-RH_{c}p_{\mathrm{sat}}\right)}{RT_{c}},C_{\mathrm{in}}^{H_{2}O}=\frac{RH_{a/c}p_{\mathrm{sat}}}{RT_{a/c}}$$

The boundary conditions for the charge conservation equation are given by Equations (25) and (26), which set the anode side of the bipolar plate as the zero-reference potential point and the cathode side with the desired loaded current density, *I*. Since the proton transport process occurs only in the CLs and PEM, a zero-flux boundary is set on the outer surface of the BP. To ensure the continuity of the temperature between neighboring channels and ignore the natural convective heat dissipation to the surroundings, the heat transfer rate of the wall around the BP is set to zero.

(25) $${\left(\phi_{s}\right)}_{\mathrm{anode}\;\mathrm{wall}}=0,\;\sigma {\left(\frac{\partial \phi_{s}}{\partial n}\right)}_{\mathrm{cathode}\;\mathrm{wall}}=I$$

(26) $${\left(\frac{\partial \phi_{e}}{\partial n}\right)}_{\mathrm{wall}}=0$$

### 2.4. Numerical Method

The computational domains of isothermal and non-isothermal calculations are physically modeled, and the equations are discretized and solved using the CFD software Fluent 6.3 and user-defined functions (UDFs). The basic set of control equations for each subregion described in Section 2.2 is constructed, and the convective and diffusive terms are corrected to establish the multi-physical field coupling relationship through customized variables. The calculations of component transport, heat transfer, and electrochemical processes in the fuel cell are realized, and the boundary conditions are given according to the actual operating conditions of the fuel cell. The coupled pressure–velocity field is solved by the SIMPLE (semi-implicit method for pressure-linked equation) algorithm. The convective terms are discretized in first-order windward format, while the diffusion terms are discretized in second-order central difference format.

## 3. Experimental Verification

Before the numerical analysis, a strict grid independence verification of the 3D computational domain is required to ensure that the simulation results have both efficiency and accuracy. Three grids are set up for the independence test, and the structural parameters of the model are shown in Table 1. The condition of a loading current density of 5500 A/m^2^ and a stoichiometric ratio of 200 under the non-isothermal model is selected. As shown in Table 3, Grid 3 with the highest grid density is used as the criterion for grid-independent verification. The relative error between the voltage results and Grid 3 (3,758,670 grids) is only 0.13% when using Grid 2 (2,835,200 grids). Grid 1 has the fewest grids (15,373,350 grids), and the relative error of its voltage reaches 2.2%, with a tendency for the error to widen. Therefore, Grid 2 is chosen as the computational grid to ensure sufficient numerical accuracy while providing reasonable computational efficiency.

**Figure 2:**
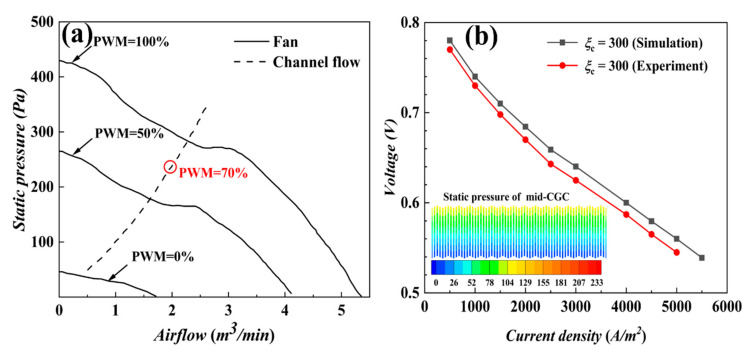
(**a**) Pressure inlet boundary value calculation based on fan characteristics; (**b**) simulation results vs. experimental data.

To verify the accuracy of the CFD model, the calculated results were compared with the experimental test results. The experiments were conducted based on the following operating conditions: 99.999% pure dry hydrogen, operating temperature of 25 ± 3 °C, relative humidity of 50 ± 10%, and air pressure of atmospheric pressure. A Sanyo DC 12 V fan was used for the fan. Figure 2 shows the fan air pressure–flow rate relationship curve [25] and experimental verification results. Under the condition of a stoichiometric ratio of 300, the pressure difference between the inlet and the outlet of the cathode flow channel is 233 Pa, which is consistent with the value of static pressure under 70% of fan PWM in Figure 2a, and the value of the air flow rate is 2 m^3^/min. As shown in Figure 2b, the numerically calculated curves match well with experimental test results. This shows that the established steady-state numerical model is accurate and reliable.

## 4. Results and Discussion

### 4.1. Isothermal Calculation

There are two mechanisms affecting the water distribution within MEA during the operation of AC-PEMFC, the blowing of water on the MEA surface by high-flow air flow, and the MEA water–water vapor dynamic equilibrium within MEA, which is closely related to the temperature. To independently explore the relative importance of these two factors, air flow rate and temperature control, in the performance of AC-PEMFC, an isothermal model was used for univariate analysis, comparing the working conditions with different stoichiometric ratios and temperatures, respectively, and analyzing the effects on the performance of the cells one by one, in combination with the results of the reactants, the water content, and the current density.

#### 4.1.1. Performance of AC-PEMFCs at Different Stoichiometric Ratios under Isothermal Conditions

**Figure 3:**
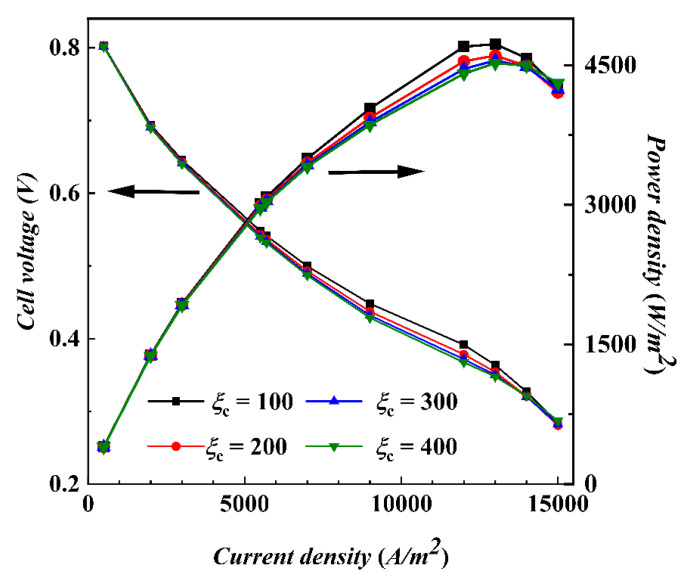
Polarization curves at different cathode stoichiometric ratios by isothermal calculation.

Figure 3 shows the curves of output voltage and power density with current density at isothermal 328 K with different cathode stoichiometric ratios. At low current density, the output power density increases dramatically with the increase in current, but the performance difference between different stoichiometric ratios is not obvious, indicating that the performance of the fuel cell is almost unaffected by stoichiometric ratios in the low current density range. The electrochemical reaction can be carried out effectively under each stoichiometric ratio condition without reaching the limiting conditions of the reaction. At high current densities, the voltage difference between different stoichiometric ratios increases gradually. The curves with a stoichiometric ratio of 100 are significantly higher in voltage and power density than other stoichiometric ratios, verifying that low stoichiometric ratios are beneficial to reduce the blowing effect and keep the MEA humidity, thus reducing the ohmic polarization loss. In addition, the curves with stoichiometric ratios of 300 and 400 are very close to each other, indicating that further increasing the stoichiometric ratio has a diminishing effect on the performance enhancement. The output reaches its maximum power when the current density reaches 13,000 A/m^2^, and the output power decreases when the current density continues to increase. Possible reasons for this include ohmic polarization due to low water content and concentration polarization due to insufficient concentration of reactants, which will be discussed later. The performance of a stoichiometric ratio of 400 is slightly higher than that of the other stoichiometric ratios after the current density reaches 14,000 A/m^2^, indicating that increasing the stoichiometric ratio can increase the reactant concentration to a certain extent at high current densities, while the variability in the effect of the different stoichiometric ratios on the water evaporation and emission gradually decreases.

**Figure 4:**
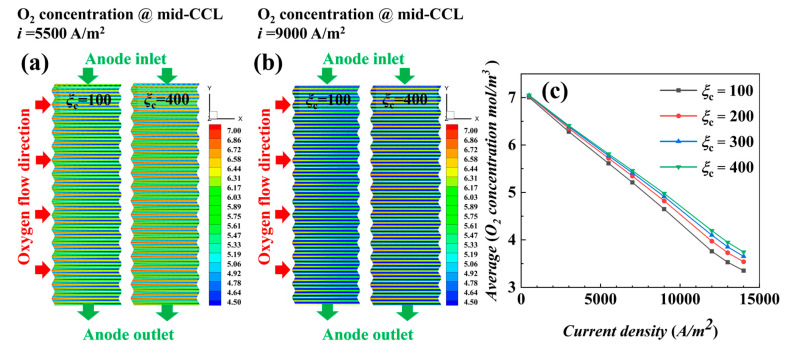

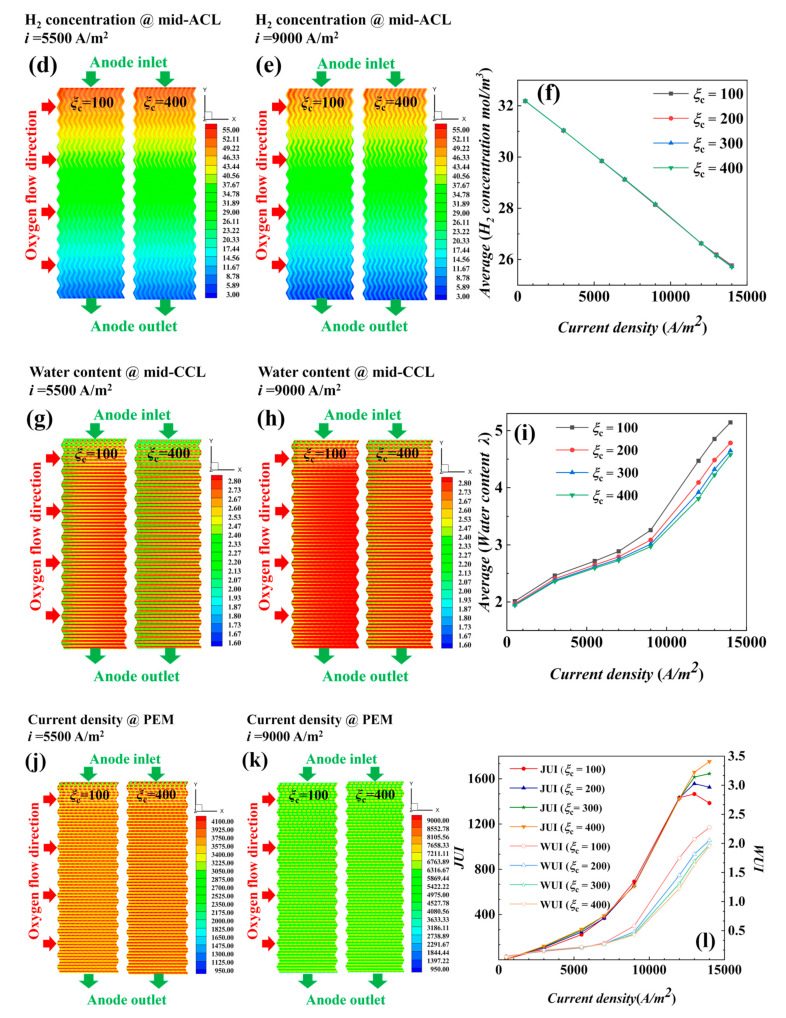
Distribution and average value at different stoichiometric ratios for *i* = 5500 A/m^2^ and *i* = 9000 A/m^2^: (**a**–**c**) oxygen concentration, (**d**–**f**) hydrogen concentration, (**g**–**i**) water content, (**j**,**k**) current density, and (**l**) uniformity index of current density and water content.

To have a more intuitive and clear understanding of the effect of stoichiometric ratio on cell performance, the distribution plots of oxygen concentration (at mid-plane of CCL), MEA water content (at mid-plane of CCL), and current density (at mid-plane of PEM) with different stoichiometric ratios were compared. The relationship curves of each parameter and with the current density are shown in Figure 4. The distribution plots of the loaded current densities *i* = 5500 A/m^2^ and *i* = 9000 A/m^2^ were selected as examples for the analysis.

The oxygen concentrations at different stoichiometric ratios for the two loading current densities in Figure 4a,b show that the oxygen concentration in the under-ridge region is slightly lower than that in the channel region, and the concentration gradually decreases along the direction of air flow (x-axis). The oxygen concentration is higher for a stoichiometric ratio of 400 than for stoichiometric ratio of 100, and the concentration is lower for *i* = 9000 A/m^2^ due to faster oxygen consumption. The average concentration of oxygen in Figure 4c gradually decreases with the increase in current density, but the oxygen concentration is generally high and sufficient; with the increase in current density, the oxygen consumption is relatively faster, and the oxygen concentration of a stoichiometric ratio of 100 is significantly lower than that of the other stoichiometric ratios at high current densities. Hydrogen concentrations in Figure 4d,e both decrease rapidly along the direction of gas flow (y-axis), and similarly, the hydrogen concentration in the region below the ridge is lower than that in the region below the channel. The average hydrogen concentration in Figure 4f decreases gradually with the increase in current density, and there is no significant difference between the curves under different stoichiometric ratio conditions, indicating that the cathode stoichiometric ratio has a very small effect on the oxidation reaction of hydrogen at the anode.

As shown in Figure 4g,h, water content at both loading current densities has a decreasing trend with an increasing stoichiometric ratio, and a stoichiometric ratio of 100 generates more water content than a stoichiometric ratio of 400. It is found from the corresponding Figure 4i plot that the increase in the horizontal mean concentration with the increase in the current density is small at low current densities, whereas the increase in the horizontal mean concentration is significantly higher at high current densities, especially at a stoichiometric ratio of 100. The increase is larger than those in other stoichiometric ratios, as shown in Figure 3, where a stoichiometric ratio of 100 has a higher output performance than a stoichiometric ratio of 400 at high current density. It is worth mentioning that, at high current density, water content is at a higher value for different stoichiometric ratio conditions, which exactly explains that the great value of power density in Figure 3 occurs not due to ohmic loss caused by the drying of MEA but due to the loss of concentration polarization caused by insufficient oxygen concentration. Under the given current density conditions, the different stoichiometric ratios in the (j) and (k) plots do not lead to significant changes in the current density distribution, which indicates that the current density distribution is mainly affected by other factors under this simulation condition.

To better grasp the current density distribution inside the fuel cell, with the help of uniformity index Equations (21) and (22), the current density uniformity index *JUI* and water concentration uniformity index *WUI* are introduced; as shown in Figure 4l, the low stoichiometric ratio has a high uniformity index of the water content with poor uniformity, but the distribution is more uniform in the current density plot, and the opposite is true for the high stoichiometric ratio. Combined with the (g) and (h) figures, it is found that a low stoichiometric ratio favors the increase in water content under the channel, which has a smaller effect on the under-ridge region, and thus, on the contrary, the uniformity is poor, similar to the results in [24]. It could be inferred that good water management can significantly affect the uniformity of current density. The distribution of cell current density is mainly influenced by the distribution of cathode oxygen and water content, which determine the reaction over-potential and proton conductivity in the cell, respectively. When loading higher current densities (here *i* = 13,000 A/m^2^, which is close to the mass transport limit), the performance loss of the cell mainly originates from the reactant transport-limited concentration polarization, and the distribution of the oxygen concentration becomes the main factor determining the current density distribution. As a result, the decreasing trend in the output voltage of the stoichiometric ratio 400 can be seen to slow down in Figure 3.

In summary, different stoichiometric ratios will affect several interdependent parameters inside the fuel cell, including the utilization of the reactants oxygen and hydrogen, the management of product water content, the uniformity of current density, etc. The correct selection of stoichiometric ratios is crucial to optimize the overall performance of fuel cells.

#### 4.1.2. Performance of AC-PEMFCs at Different Temperatures under Isothermal Conditions

**Figure 5:**
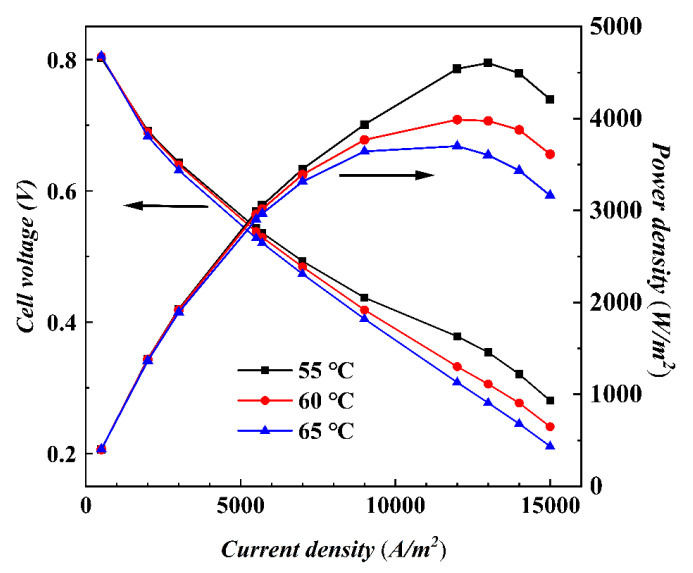
Polarization curves at different temperatures by isothermal calculation.

The output performance variation curves with increasing current density for different temperatures at a stoichiometric ratio of 200 are shown in Figure 5. Compared with Figure 3, the temperature change has a more significant effect on performance than the stoichiometric ratio changes when loading high current densities. The voltages at 55 °C are consistently higher than those at 60 °C and 65 °C, indicating that although high temperatures can increase the activity of the chemical reaction, they also promote the conversion of MEA water to water vapor, resulting in a drier MEA and a lower proton conductivity. Lower operating temperatures help to reduce the transfer of MEA water to water vapor, thus ensuring the wetting of MEA and reducing ohmic losses. As the current density increases to about 13,000 A/m^2^, the power reaches a maximum value, after which the power density decreases with a further increase in current. Therefore, the effect of temperature on the cell should not be underestimated, and the selection of the optimal operating temperature is essential to balance these effects.

**Figure 6:**
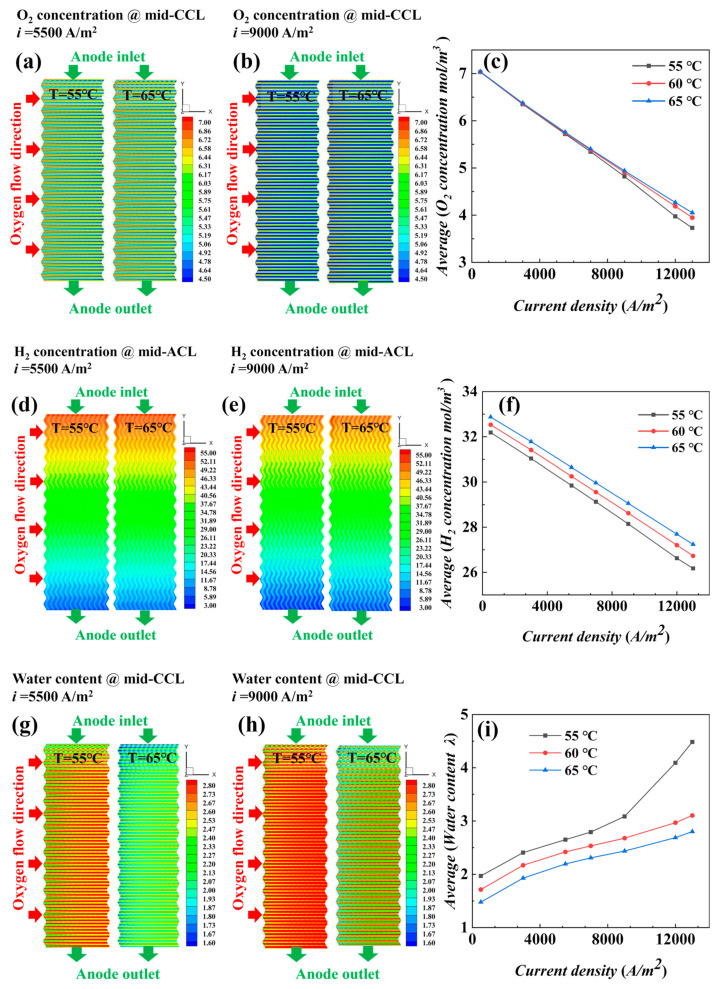

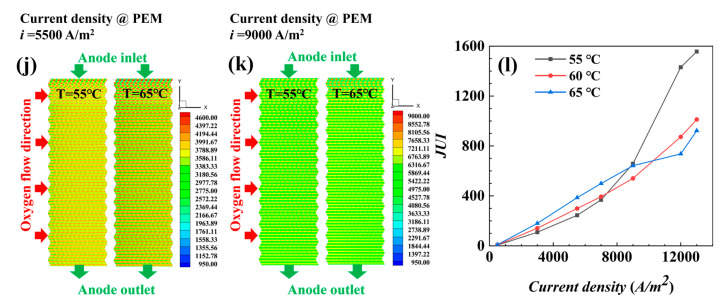
Distribution and average value at different temperatures for *i* = 5500 A/m^2^ and *i* = 9000 A/m^2^: (**a**–**c**) oxygen concentration, (**d**–**f**) hydrogen concentration, (**g**–**i**) water content, (**j**,**k**) current density, and (**l**) uniformity index of current density.

To have a clearer understanding of the effect of temperature on fuel cell performance, the distribution plots of oxygen concentration (cathode catalytic layer (CCL)), MEA water content (mid-plane of CCL), and current density (PEM) at different temperatures were compared. Additionally, the relationship curves of each parameter and the current density are shown in Figure 6. The distribution plots of the loaded current densities *i* = 5500 A/m^2^ and *i* = 9000 A/m^2^ were selected as examples for the analysis.

As shown in Figure 6a,b, the effect on the distribution of oxygen concentration at different temperatures is small. In Figure 6c, the average concentration of oxygen at three different temperatures does not differ significantly at a given current density. As the current density increases, the decreasing trend of oxygen concentration at three temperatures begins to diverge, the oxygen concentration at 55 °C decreases the most, and the oxygen concentration at 65 °C is high because of the decrease in reaction polarization loss when the temperature increases, which, combined with Equations (10) and (14), are reflected in the increase in temperature; *j*_c_ decreases; *S*_c_ decreases; and less oxygen is consumed, so the concentration becomes higher. Similar to the oxygen concentration in Figure 6f, lower temperatures increase the reaction polarization loss, which, in combination with Equations (10) and (14), is reflected in the fact that as temperature decreases, *j*_c_ increases, *S*_c_ increases, and more hydrogen is consumed, and therefore, the hydrogen concentration is significantly lower than the other temperature conditions, even at low current densities.

The water content in Figure 6g,h increases gradually along the x-direction, and the water content is higher overall when *i* = 9000 A/m^2^, where it is significantly higher at 55 °C than at 65 °C. In Figure 6i, the lower the temperature, the higher the average value of water content, which is more significant at high current density, because the water produced in the catalytic layer mainly exists in the form of MEA water, and the conversion between MEA water and water vapor is in dynamic equilibrium. The increase in temperature accelerates the conversion of MEA water to water vapor, and more water vapor is taken out of the stack under the effect of blowing. Additionally, under the low temperature condition, the evaporation rate of the water is low, which is conducive to the retention of water content. In Figure 6j,k, under low and medium loading density, a current density value of 65 °C is in a higher state than that of 55 °C, but under high current density, it is the opposite, which is related to the current density distribution in Figure 6l; a current density distribution of 55 °C is more homogeneous than that of 65 °C in the region of low and medium loading currents, but in the region of high currents, the uniformity of a current density distribution of 65 °C is good.

### 4.2. Non-Isothermal Calculation

To explore the distribution characteristics of the parameter under actual operation, a non-isothermal model was used. This model compares different stoichiometric ratio conditions and analyzes temperature homogeneity, current density homogeneity, and water content homogeneity to find the optimal stoichiometric ratio.

**Figure 7:**
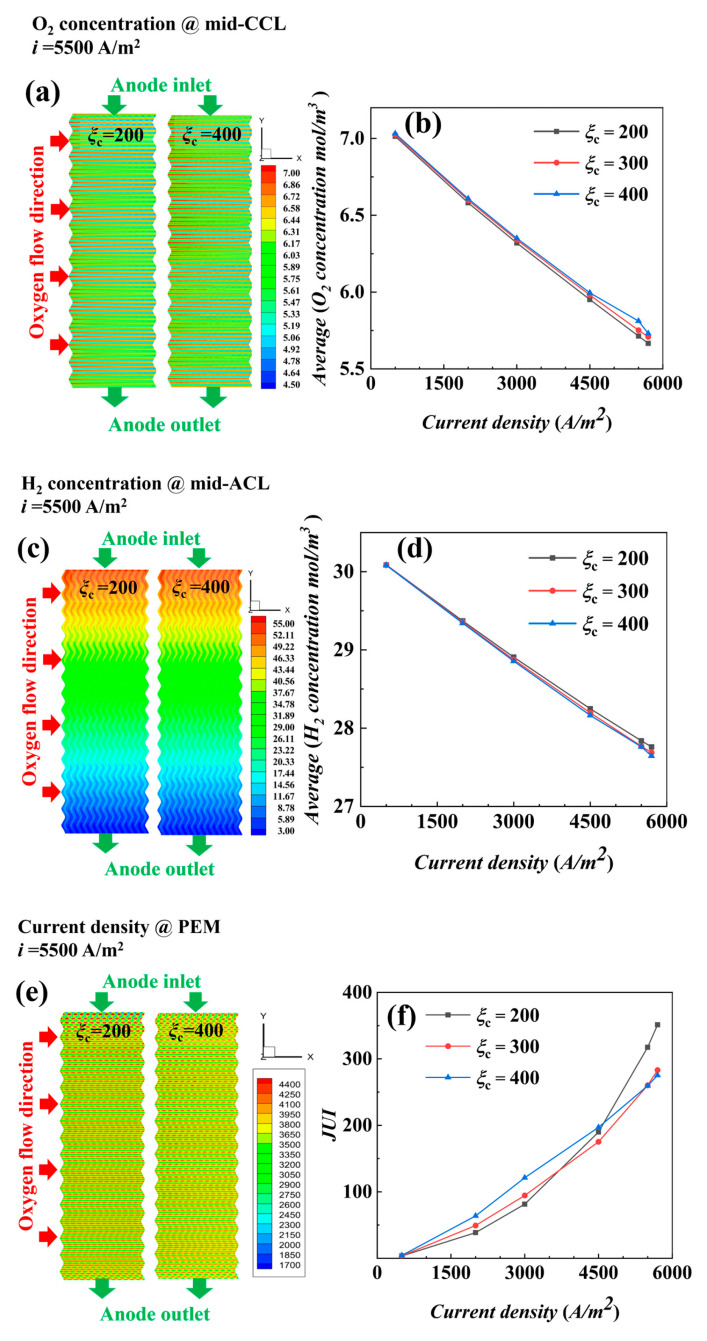

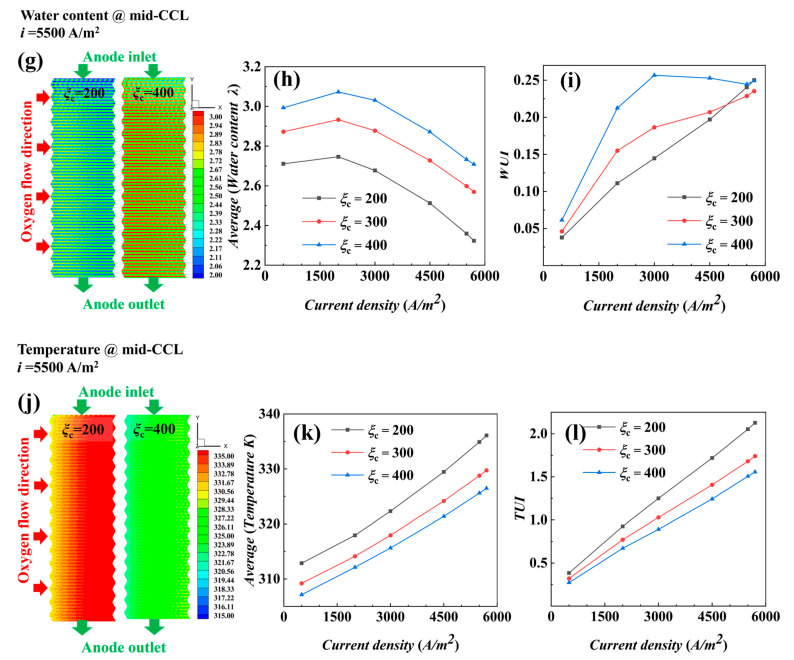
Distribution and average value at different stoichiometric ratios for *i* = 5500 A/m^2^: (**a**,**b**) oxygen concentration, (**c**,**d**) hydrogen concentration, (**e**,**f**) current density, (**g**–**i**) water content, and (**j**–**l**) temperature.

The distributions of oxygen concentration (mid-plane of CCL), hydrogen concentration (mid-plane of ACL), water content (mid-plane of CCL), temperature (mid-plane of CCL), and current density (proton exchange membrane) distributions and the variation graphs obtained by solving the physical fields and reaction properties in the fuel cell at stoichiometric ratios of 200 and 400 with *i* = 5500 A/m^2^ are shown in Figure 7.

Oxygen concentration in the under-ridge region is slightly lower at each condition in Figure 7a, while oxygen concentration in the under-channel region is slightly higher, and all of them show a trend of gradually decreasing concentration along the direction of air flow. The average concentration of oxygen with different stoichiometric ratios in Figure 7b decreases gradually with the increase in current density, and the oxygen concentration with a stoichiometric ratio of 200 is the lowest, but the oxygen is still relatively sufficient. Hydrogen concentration gradually decreases along the (-y) axis in Figure 7c, and the average concentration of hydrogen at the three stoichiometric ratios decreases linearly with increasing current density in Figure 7d.

The water content in Figure 7g increases with the increase in stoichiometry in all cases, and the water content of MEA in the ridge region is higher than that in the channel region at a small stoichiometric ratio of 200, indicating that the amount of water discharged from the channel region is larger under the effect of blowing. The water content is higher on the air inlet side and decreases gradually along the x direction to the outlet side. In Figure 7h the increases and then decreases with the increase in current density, indicating that, at low current density (<1000 A/m^2^), the water production is low, and the blowing effect of a large air flow further reduces the water content of MEA. When the current density is >2000 A/m^2^ the key factor affecting the water content is temperature rather than the blowing effect, which verifies the conclusion of the previous isothermal analysis.

To better grasp the distribution of water content inside the fuel cell, with the help of the uniformity index Formulas (21) and (22), the distribution curve of the water uniformity index *WUI* is obtained as in Figure 7i; the uniformity of water under different stoichiometric ratios is different, and a stoichiometric ratio of 200 shows the best water distribution uniformity under low current density. Additionally, with the increase in current density, the uniformity index gradually increases, while the uniformity gets worse and worse. The water uniformity index of stoichiometric ratios 300 and 400 is high and poor at low densities, and with the increase in current density, the increase in its water uniformity index slows down, and even at densities of 5500 A/m^2^, the uniformity index of a stoichiometric ratio of 300 is lower than that of a stoichiometric ratio of 200.

In Figure 7j, temperature change distribution graphs visualize that, with the increase in stoichiometric ratios, temperatures get lower and lower, and the temperature of water distribution is lower and lower at the *i* = 5500 A/m^2^ condition; the temperatures are all higher than *i* = 3000 A/m^2^. As the reaction proceeds, the temperature inside the cell rises, and the temperature of a stoichiometric ratio of 200 is higher than those of the other two stoichiometric ratios, and a stoichiometric ratio of 400 means that there is more gas flow to help with heat removal, providing better cooling. This finding further validates the conclusion of Zhang et al. [20] that an increase in the stoichiometric ratio favors heat emission. The average value of the temperatures in Figure 7k rises gradually with the increase in current density, with a stoichiometric ratio of 400 having the best temperature uniformity. It also confirms the findings of Wei et al. [21], who found that increasing the air flow rate can effectively improve the temperature uniformity in the reactor. In Figure 7e, under low electric density, a high stoichiometric ratio produces high current density, but under the condition of *i* = 5500 A/m^2^, the difference of a stoichiometric ratio on electric density is not significant; however, the uniformity of electric density is different in Figure 7f, the uniformity of electric density is the same in the initial stage, and the gap is gradually widened with the increase in electric density. The electric density uniformity correlates with the uniformity of water content and the uniformity of temperature. It can be seen that the trend of current density uniformity is consistent with water content uniformity at low electric density, while at high electric density, it is consistent with temperature uniformity.

**Figure 8:**
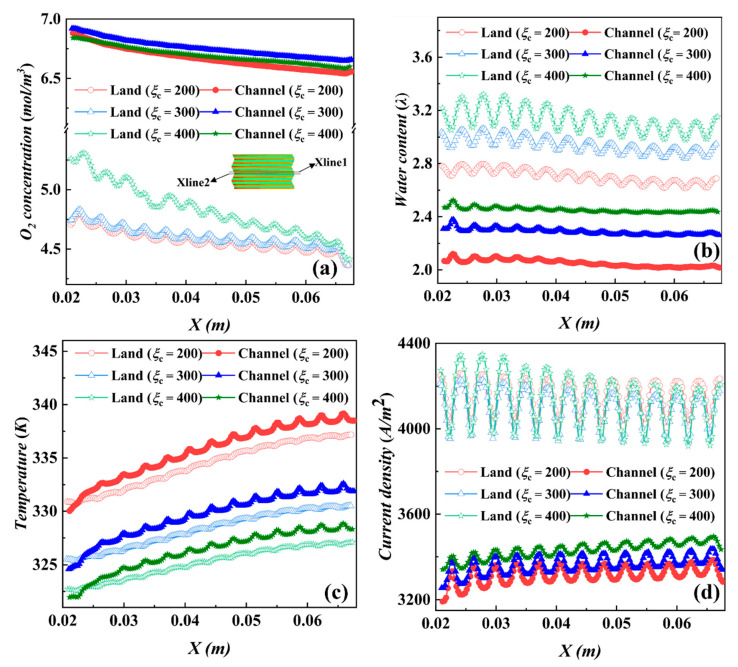
Distribution along the monitoring lines XLine1 (land) XLine2 (channel) at different stoichiometric ratios for *i* = 5500 A/m^2^: (**a**) oxygen concentration, (**b**) water content, (**c**) temperature, and (**d**) current density.

In Figure 7a,i, when the oxygen and water content uniformity increases from *i* = 5500 A/m^2^ to 5700 A/m^2^, the fluctuation of a stoichiometric ratio of 400 is different from the trend of other stoichiometric ratios. Further analysis of the internal distribution of ridges and channels along the monitoring lines XLine1 and XLine2 at *i* = 5500 A/m^2^ reveals the distribution curves of oxygen, water content, temperature (cathode catalytic layer center plane XY plane), and current density (PEM plane XY plane) along the XLine1 and XLine2 ridges and channels under different stoichiometric ratios, as shown in Figure 8.

There is little difference in oxygen content in the channel region for the three stoichiometric ratios in Figure 8a, but a ridge region stoichiometric ratio of 400 contains more oxygen than the other stoichiometric ratios, mainly because the large air flow rate intensifies the convective mass transfer process of the gas from the channel down to the ridge. Higher stoichiometric ratios in Figure 8b,c are associated with more water content, and conversely, higher stoichiometric ratios are associated with lower temperatures; furthermore, the ridge region contains more water than the channel region, while the temperature is slightly lower than the channel region temperature.

In Figure 8d at *i* = 5500 A/m^2^, the ridge region with a stoichiometric ratio of 200 has the highest electric density, and the combination of reactant concentration, water content, and temperature distribution characteristics reveals that the higher water content in this region is the key factor. A comparison of the current densities in the channel region shows that the current density in the channel region with a stoichiometric ratio of 200 is lower, and the main reason for this is also determined by the low water content. At high current densities, the reaction rate and heat generation increase, which leads to water loss in the channel region, even though a large stoichiometric ratio flow removes more heat. The effects of temperature increase and water loss are mutually reinforcing and unbalanced states. Once the temperature is too high, the accelerated water loss will lead to drier MEA; therefore, the high-temperature condition should be avoided as much as possible. In addition, at *i* = 5500 A/m^2^, each parameter of the ridge region for the three stoichiometric ratios fluctuates in a larger range along the x-axis (direction of air flow), which is related to the arrangement of the hydrogen channel ridges at the anode side.

**Figure 9:**
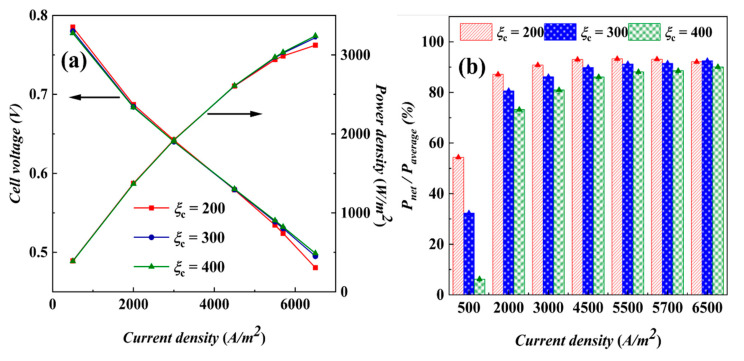
Performance results by non-isothermal calculations: (**a**) polarization curves and (**b**) net power relative values.

Figure 9a shows the variation curves of output voltage and power density with current density for different cathode stoichiometric ratios at non-isothermal temperatures. At the initial stage of loading current density, the initial voltages of fuel cells corresponding to different stoichiometric ratios are different, and the cell with a stoichiometric ratio of 200 has the highest voltage, which indicates that, at lower stoichiometric ratios, the internal resistance of the cell is lower, and the cell’s initial working condition is more ideal; with the increase in current density, the voltages at all stoichiometric ratios show a decreasing trend, which indicates that the polarization loss increases with the increase in current density, and is due to the combined effect of ohmic loss and concentrated polarization. The voltage decreases the fastest at a stoichiometric ratio of 200, and the decreasing trend at stoichiometric ratios of 300 and 400 is not significant and the difference is not large.

An AC-PEMFC system consisting of 30 cells and 2 cooling fans is taken as an example to analyze the net power in actual operation. The fan power corresponding to each stoichiometric ratio is listed in Table 4. The relative value of the net power *P*_net_ of the power stack after considering the consumption of the fan power is shown in Figure 9b. It is found that when the current density is less than or equal to 5700 A/m^2^, the net power of a stoichiometric ratio of 200 is the highest, and the performance of a stoichiometric ratio of 300 is the highest in high density; combined with the distribution characteristics of each component in Figure 7, the cell performance is optimal at a stoichiometric ratio of 300.

## 5. Conclusions

To clarify the interaction of air flow rate and temperature in the performance of AC-PEMFC, this study constructed a full-scale multi-physics coupling numerical model of AC-PEMFC. A homogeneity index was introduced to explore the influence of a key parameter distribution on fuel cell performance. Both isothermal and non-isothermal models were used to carry out univariate and multivariate analyses, resulting in the following main conclusions:

(1) The air supplied by fans is sufficient, and the cathode stoichiometric ratio has little effect on the concentration of reactants and water vapor. Low stoichiometric ratios at high current densities show slightly better water content and distribution uniformity, leading to better output performance.
(2) The temperature is the key factor affecting water content and output power. As current density increases, lower temperature conditions help maintain MEA humidity and improve the uniformity of current density distribution, positively impacting cell performance.
(3) Under actual operating conditions, the performance of AC-PEMFC is affected by multiple factors. An increase in temperature above the operating range usually leads to performance deterioration. The difficulty of heat dissipation in the channel region further aggravates performance degradation. However, increasing the stoichiometric ratio can significantly enhance heat dissipation and improve water content and temperature uniformity, resulting in a more homogeneous electric density distribution. This study found that the optimal fuel cell performance is achieved with a stoichiometric ratio of 300. The proper selection of a stoichiometric ratio is essential in designing and operating AC-PEMFC to ensure optimal performance under various operating conditions.

In summary, this study exhaustively analyzes the effects of air flow rate and temperature on fuel cell performance by constructing a simulation model of AC-PEMFC. It reveals the importance of temperature management and stoichiometric ratio in optimizing cell performance. Additionally, by introducing the uniformity index, the study accurately grasps the distribution of component concentrations inside the cell. It confirms that the uniformity of water vapor content and temperature distribution can be effectively improved under the condition of an appropriate stoichiometric ratio (300), thus optimizing the overall performance. These findings provide a significant theoretical basis for the design and operation of fuel cells and guidance for achieving a more efficient and reliable air-cooled fuel cell system. For future research, it is of great practical significance to study the long-term operation stability and the mechanism of MEA degradation.

## Figures and Tables

**Table 1:** Structural parameters and operating conditions.

Parameter	Value
Cathode channel length (mm)	60
Anode channel depth (mm)	0.35
Anode channel width(mm)	1
Cathode channel depth (mm)	2
Depth of cathode channel (mm)	1
Thickness of GDL (mm)	0.015
Thickness of PEM (mm)	0.01
Anode/cathode stoichiometric ratio	1.2/100, 200, 300, 400
Anode/cathode runner inlet temperature (K)	301/301
Anode/cathode relative humidity (%)	0/50
Anode/cathode operating pressure (MPa)	0.15/0.1 (ABS)

**Table 2:** Electrochemical and transport parameters of the AC-PEMFC model.

Parameter	Value
Porosity of porous layers	$\varepsilon_{GDL}/\varepsilon_{CL}=0.6/0.5$
Ionomer volume fraction of ACL/CCL	$\varepsilon_{m}=0.2$
Volumetric reference current density (A/m^3^)	$a_{0}i_{0,a}/a_{0}i_{0,c}=10^{9}/10^{4}$
Transfer coefficient	$\alpha_{a}=\alpha_{c}=1$
Electro-osmotic drag coefficient [33]	$n_{d}=2.5\lambda /22$
GDL/MPL/CL permeability (m^2^) [33]	$K_{GDL}/K_{CL}=1\times 10^{-12}/1\times 10^{-13}$
BP/GDL/MPL/CL electrical conductivity (S/m)	$\sigma_{BP/}\sigma_{GDL\;/}\sigma_{CL=10000/1250/300}$
Ionic conductivity of ionomer (S/m)	$\kappa =\left(0.5139\lambda -0.326\right)\exp\left[2222\left(\frac{1}{303}-\frac{1}{T}\right)\right]$
Thermal conductivity (W/m·K) [40]	$k_{BP}/k_{GDL}/k_{CL}/k_{PEM}=135/1/1/0.95$
Gas diffusivity	$D^{H_{2}}/D^{O_{2}}/D^{H_{2}O}=1.1\times 10^{-4}/3.2\times 10^{-5}/7.35\times 10^{-5}$
Diffusivity of MEA water (m^2^/s) [41]	$D_{m}^{H_{2}O}=\left\{\begin{array}{c} 5.93\times 10^{-5}\lambda (e^{0.28\lambda }-1)e^{\left(-4269/T\right)}\\ 7.97\times 10^{-6}\lambda (1+161e^{-\lambda })e^{\left(-4269/T\right)} \end{array}\right.\begin{array}{c} if\;0<\lambda \leq 3\\ otherwise \end{array}$
Dry MEA density (kg/m^3^)	$\rho_{PEM}=1980$
MEA equivalent weight (kg/mol)	EW=1.1

**Table 3:** Grid independence results.

	Number of Grids	Current Density *i* = 3000 A/m^2^	
		Output Voltage (V)	Relative Error
Grid 1	1,537,350	0.52310	2.2%
Grid 2	2,835,200	0.53461	0.13%
Grid 3	3,758,670	0.53531	/

**Table 4:** Fan power at different PWMs.

*ξ_c_*	Static Pressure (Pa)	PWM	Fan Power (W)
200	152	45%	24.34
300	233	70%	35.64
400	363	100%	49.2

## Data Availability

Data are contained within the article.
